# Analysis of the intensity of immune cell infiltration and immunoreactivity of RCAS1 in diffuse large B-cell lymphoma of the palatine tonsil and its microenvironment

**DOI:** 10.1007/s00441-015-2157-0

**Published:** 2015-03-17

**Authors:** W. Kazmierczak, A. Lazar, R. Tomaszewska, T. J. Popiela, K. Koper, Lukasz Wicherek, M. Dutsch-Wicherek

**Affiliations:** 1 Department of Otolaryngology and Oncological Laryngology with Subdivision of Audiology and Phoniatry, Jurasz’s University Hospital, Bydgoszcz, Poland; 2 Department of Pathology, Jagiellonian University, Kraków, Poland; 3 Department of Radiology, Jagiellonian University, Kraków, Poland; 4 Department of Gynecology and Oncology, The Lukaszczyk Oncological Center, Bydgoszcz, Poland; 5 Chair of Gynecology, Oncology and Gynecological Nursing, Ludwik Rydygier Collegium Medicum in Bydgoszcz, Nicolaus Copernicus University, Bydgoszcz, Poland; 6 Department of Pediatric Otolaryngology, Jagiellonian University Children’s Hospital in Krakow, Kraków, Poland

**Keywords:** RCAS1, Diffuse large B-cell lymphoma, Palatine tonsils, Tumor microenvironment, Human

## Abstract

Non-Hodgkin lymphoma of Waldeyer’s ring constitutes a small percentage of cases of palatine tonsil malignancies and its precise etiology remains unknown. RCAS1 (receptor cancer-binding antigen expressed on SiSo cells) has been demonstrated to be associated with poor prognosis, the development of lymph node metastases and participation in tumor microenvironment remodeling. Our aim is to analyze the potential role of RCAS1 expression in the tumor and tumor microenvironment in the development of early-stage palatine tonsil B-cell lymphomas. We selected 20 patients and analyzed tissue samples from the lymphoma and tumor microenvironment of each patient and from a reference group of 20 patients with chronic tonsillitis. The presence of RCAS1 protein immunoreactivity was demonstrated in 65 % of the examined tissue samples of diffuse large B-cell lymphoma and in 25 % of the analyzed stromata in which it was exhibited by CD68-positive cells identified as macrophages and dispersed throughout the stroma. RCAS1 immunoreactivity in the lymphoma tissue samples remained at a level comparable with that of the reference and was significantly higher in these samples than in those from the stroma. Chronic inflammation of the palatine tonsils thus results in intensive infiltration by various types of immune system cells and in excessive RCAS1 immunoreactivity, both of which confirm the important regulatory role of RCAS1 in the immune response in the mucosa-associated lymphatic tissue of Waldeyer’s ring. RCAS1 seems to be involved in creating tumor-induced inflammation in the tumor and its microenvironment.

## Introduction

Extra-nodal head and neck lymphomas are rare conditions and the tonsils and sinuses are the most common sites of localization (Laskar et al. 2007; Jacobs et al. 1986; Yamanaka et al. 1985; Hart et al. 2004). Non-Hodgkin lymphoma of Waldeyer’s ring constitutes a small percentage of cases of palatine tonsil malignancies and its precise etiology remains unknown. Infectious organisms, including the Epstein-Barr virus (EBV) and human immunodeficiency virus, are increasingly recognized as possible causes (Laskar et al. 2007; Hart et al. 2004). The most common type of lymphoma found in the palatine tonsils is the B-cell lymphoma, with DLBCL (diffuse large B-cell lymphoma) predominating (67–96 % of cases). The majority of these cases represent loco-regional instances of the disease (stages I-II; Laskar et al. 2007; Jacobs et al. 1986; Yamanaka et al. 1985; Hart et al. 2004).

The tumor microenvironment is the tissue that supports the tumor’s growth (Witz 2009). In our previous studies, we analyzed and demonstrated the involvement of the tumor microenvironment in the development of various types of malignancies, including uterine cervical carcinoma, ovarian cancer and pharyngeal squamous cell carcinoma, all of which are associated with the presence of a suppressive tumor microenvironment ( Dutsch-Wicherek 2010; Dutsch-Wicherek and Kazmierczak 2013; Dutsch-Wicherek et al. 2010, 2012, 2013; Walentowicz-Sadlecka et al. 2013; Jozwicki et al. 2011; Galazka et al. 2012; Wicherek et al. 2012).

RCAS1 (receptor cancer-binding antigen expressed on SiSo cells) is expressed by various human cancer cells and during the induced apoptosis of T and B lymphocytes and native killer (NK) cells and thus participates in tumor escape from host immunological surveillance. In many cases involving various types of malignant neoplasms, RCAS1 has been demonstrated to be associated with poor prognosis, the development of lymph node metastases and participation in tumor microenvironment remodeling (Dutsch-Wicherek et al. 2009, 2010, 2012; Sonoda et al. 1996, 2005, 2006, 2008, 2009; Sonoda 2011). RCAS1-positive macrophages have been identified in the cancer microenvironment of patients suffering from pharyngeal and laryngeal squamous cell carcinomas and are associated with the presence of lymph node metastases (Dutsch-Wicherek et al. 2009, 2012; Dutsch-Wicherek 2010). The expression of RCAS1 by the tumor cells and tumor-associated macrophages might help to create the immunosuppressive microenvironment in patients with parotid gland adenocarcinoma, pharyngeal and laryngeal cancer, ovarian cancer, uterine cancer and other types of malignant neoplasms (Dutsch-Wicherek 2010; Dutsch-Wicherek and Kazmierczak 2013; Dutsch-Wicherek et al. 2010, 2012, 2013; Walentowicz-Sadlecka et al. 2013; Jozwicki et al. 2011; Galazka et al. 2012; Wicherek et al. 2012; Sonoda et al. 1996, 2005, 2006, 2008, 2009; Sonoda 2011).

The physiological role that RCAS1 of the palatine tonsils plays in the cell regulaton and pathology of the immune system, in B-cell lymphoma and in lymphatic system-originating malignancies is of interest. RCAS1 expression has been observed in all studied tissues of the palatine tonsils and adenoids, with the most intensive expression being found in the reticular epithelium of the crypts and in the reactive centers of the lymphatic follicles (Dutsch-Wicherek et al. 2005). The essential regulatory role of RCAS1 in controlling erythroid progenitor cell maturation has been established. Erythroid progenitor cells have putative receptors for RCAS1 (RCAS1-R); high RCAS1-R expression has been found in the cells during the early stages of erythropoietic cell differentiation but its expression fades with cell maturation. The lowest level of production of RCAS1 has been identified in resting macrophages in which it nevetheless increases following lipopolysaccharide (LPS) stimulation. The cytolysis of progenitor cells has been observed when these cells are co-cultured with LPS-activated macrophages. Consequently, macrophages have been suggested to regulate human erythropoiesis negatively through RCAS1 expression (Matsushima et al. 2001; Suehiro et al. 2005). Moreover, RCAS1 regulation has been demonstrated to play an important role in creating maternal immune tolerance during pregnancy (Wicherek 2009; Galazka et al. 2008; Wicherek et al. 2007; Tskitishvili et al. 2010). Thus, RCAS1 might also play an important role in lymphatic-system-originating pathologies.

The aim of the present study is to analyze the potential role of RCAS1 expression in the tumor and tumor microenvironment in the development of early-stage palatine tonsil B-cell lymphomas.

## Materials and methods

For our study, we recruited patients with the most common types of non-Hodgkin lymphoma occurring in adults, namely, tonsillar DLBCL and tonsillar DLBCL with cervical lymph node involvement (i.e., stages I and II of the disease). We selected 20 patients and analyzed tissue samples from the lymphoma and tumor microenvironment of each paatient. The tumor microenvironment or stroma was defined as the surrounding tissue with an area of 1 cm^2^ macroscopically and histologically free of malignant infiltration and with the distance from the tumor front not exceeding 1 cm. The patient’s consent was obtained in each case. Additionally, approval for the research program was granted by the Ethical Committee of the Jagiellonian University in Krakow (KBET/90/B/2005). All the tissue samples were histopathologically verified. Following the fixation of the surgically removed material in formalin, pathological analysis with classical hematoxylin and eosin staining techniques was performed in the Pathology Department of the Jagiellonian University by two experienced pathologists (R.T. and A.L.) working independently. First, the tissue was fixed in a solution of 10 % formalin and then rinsed, dehydrated and transferred through a progressively increasing concentration of ethanol (from 50 % to absolute alcohol) and a series of xylenes (I–III) to molten paraffin wax. Finally, the tissue blocks were sectioned and the resulting sections placed onto glass slides. The process was mainly automated; however, both the paraffin embedding and the cutting of the tissue samples into 3– to 4-μm-thick sections were performed manually.

The characteristics of the patient group are presented in Table 1.

**Table 1:** Patient group with non-Hodgkin lymphoma

Malignant non-Hodgkin B cellular lymphoma	Number/age
All patients	20
Male	12
Female	8
Age range (average) of all patients in years	16–84 (62)
Age range (average) of males in years	43–81 (63.8)
Age range (average) of females in years	16–84 (59.4)

### Reference group

As a reference group, we chose to collect palatine tonsils that had been removed from patients suffering from recurrent tonsillitis (Table 2). In these tissue samples, we evaluated both the epithelium lining of the tonsils and the lymphoid tissue.

**Table 2:** Reference group with recurrent tonsillitis

Palatine tonsils	Number/age
All patients	20
Male	12
Female	8
Age range (average) of all patients in years	14–56 (32.6)
Age range (average) of amles in years	14–56 (34.41)
Age range (average) of females in years	21–54 (29.87)

### Immunohistochemical analysis

In the present study, we analyzed the immunoreactivity levels of various antigens in the palatine tonsil lymphoma and its stroma. We also aimed to evaluate the distribution of the antigen immunoreactivity throughout the tissue of the tumor, including its stroma. For this reason, we chose the immunohistochemical method for our study. This is also the only method that shows the actual architecture of the interaction between the tumor and its stroma. The immunohistochemical expression of RCAS1, CD3, CD25, CD68, CD69 and Foxp3 antigens was performed in the Pathology Department of the Jagiellonian University. From each case, sections of 5 μm in thickness were placed on slides and stained to visualize RCAS1-, CD3-, CD25-, CD68-, CD69- and Foxp3-positive cells. In all cases, immunohistochemistry was performed by applying the Envision method with Dako Autostainer. The samples were stained automatically by immunohistochemical staining based on the antigen-antibody reaction. Microscopy was performed with an Axio Zeiss microscope and the tissue sections were evaluated under both 20× and 40× magnification. The following antibodies were applied: mouse monoclonal antibody Anti-RCAS1 (Medicaland Biological Laboratories, Naka-ku Nagoya, Japan; in DAKO Antibody Diluent with Background Reducing Components; DAKO, Denmark; dilution 1:1000), monoclonal mouse antibody ImmunOTM (MP Biomedicals; clone 1A12; dilution 1:1000), CD68 (DAKO, clone PG-M1; dilution 1:50), CD3 (Novocastra, clone IF6; dilution: 1:50), CD25 (interleukin-2 Receptor, NCL-CD25-305; Novocastra; dilution 1:25) and Foxp3 according to the manufacturer’s instructions. Visualization of the reaction products was then performed by using AEC (3-amino-9-ethyl-carbazole) as a chromogen (AEC Substrate Chromogen ready-to-use; DAKO, Denmark) for 10 min at room temperature. Sections were counterstained with hematoxylin and mounted in glycergel. As a positive control, a tonsil specimen was taken for RCAS1 immunostaining. All staining was performed with the same procedure. The primary antibody was omitted as a negative control. RCAS1 expression in the area of the palatine tonsils in the epithelium and in the stroma was evaluated on entire slides as follows: 0 no reactivity; +1 weak and, when observed, any cytoplasmic (also granular in paranuclear region) staining (in up to 10 % of positive cells); +2 marked cytoplasmic staining (sometimes together with membranous staining in 11–30 % of the cells); +3 high expression (more than 30 % of positive cells). Variable scales were used to evaluate the number of cells semi-quantitatively, depending on their general number in the specimen. CD25+, CD3+, CD68+ and CD69+ cells were thus estimated as follows: 0 lack of positive cells; +1 single positive cells in the specimen; +2 1–5 positive cells per high power field; +3 more than 5 positive cells per high power field.

We were able to distinguish the macrophages from the stromal cells by their respective morphologies, i.e., by their characteristic size, nuclear-cytoplasmic ratio and nuclear image.

## Statistical analysis

The distribution of variables in the study groups of the patients examined with the use of the Shapiro-Wilk test showed that each of the patients was indeed different from normal. The statistical significance between the groups was determined by the Kruskal-Wallis test, with one-way analysis of variance by ranks. The Mann–Whitney *U* test was then used as applicable. All statistical analyses were carried out with the Statistica 8.0 software program. A *P*-value of <0.05 was considered as indicative of statistical significance.

## Results

In the current study, the levels of immunoreactivity of CD3, CD25, CD68, CD69, Foxp3 and RCAS1 were determined in palatine tonsil DLBCL and its microenvironment and in the tissues of the reference group, including the epithelium lining of the tonsils and the lymphoid tissue of the palatine tonsils derived from patients with chronic tonsillitis.

### Analysis of CD3 antigen immunoreactivity

CD3 antigen immunoreactivity was observed in all the lymphoma tissue samples from both the tumor and the stroma and exhibited a membrane-cytoplasmic type of expression (). CD3 antigen immunoreactivity was identified in all the lymphatic tissue samples and was also found in 75 % of the epithelial tissue samples. The immunoreactivity here also exhibited a membrane-cytoplasmic type of expression and was identified in half of the lower part of the tonsillar crypts epithelium from the chronically inflamed palatine tonsils.

### Analysis of CD25 antigen immunoreactivity

CD25 immunoreactivity was observed in 90 % of the lymphoma tissue samples and in 50 % of the stromata samples. CD25 antigen immunoreactivity was observed in all the examined samples of lymphatic tissue and tonsillar crypts epithelium. It exhibited a membrane-cytoplasmic type of expression and was most prominent in the epithelia of the tonsillar crypts.

### Analysis of CD68 antigen immunoreactivity

**Fig. 1:**
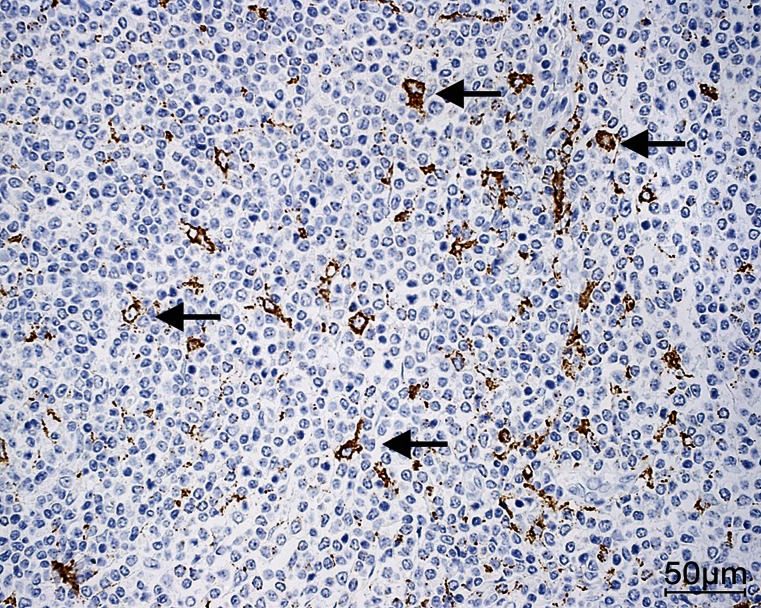
Infiltration of CD68 in both the lymphoma and its stroma (*arrows* CD68-positive macrophages). Magnification ×40. *Bar* 50 μm

CD68 immunoreactivity was present in 95 % of the lymphoma tissue samples and in 60 % of the stroma samples. CD68-positive cells infiltrating the lymphoma and its stroma are shown in Fig. 1. CD68 antigen immunoreactivity was observed in all the samples of the lymphatic tissue of the chronically inflamed palatine tonsils and in 10 % of the samples of the palatine epithelia; this immunoreactivity was the most prominent in the reticular epithelium of the crypts and exhibited a membrane-cytoplasmic type of expression.

### Analysis of CD69 antigen immunoreactivity

CD69 immunoreactivity was present in 95 % of the lymphoma tissue samples and in 70 % of the stroma samples. CD69 antigen immunoreactivity was observed in all the lymphatic tissue samples of chronically inflamed palatine tonsils, although not in the epithelia and it exhibited a membrane-cytoplasmic type of expression.

### Analysis of Foxp3 antigen immunoreactivity

Although Foxp3 immunoreactivity was not observed in the stroma of the tumor, it was present in 30 % of the lymphoma tissue samples. Foxp3 antigen immunoreactivity was observed in 90 % of the samples of lymphatic tissue from the chronically inflamed palatine tonsils but was not found in the epithelia. It occurred in lymphocytes and demonstrated a nuclear type of expression.

### Analysis of RCAS1 immunoreactivity

**Fig. 2:**
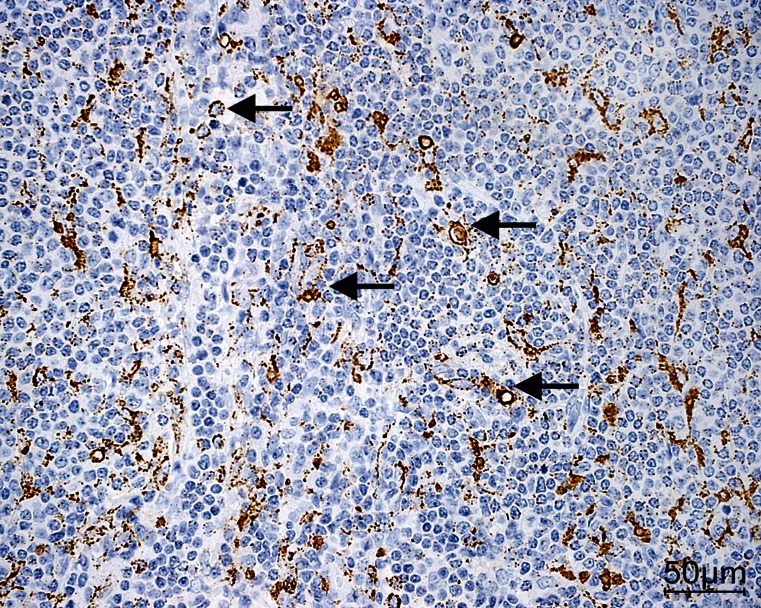
RCAS1 (receptor cancer-binding antigen expressed on SiSo cells) immunoreactivity in the lymphoma and its stroma (*arrows* RCAS1-positive macrophages). Magnification ×40. *Bar* 50 μm

RCAS1 immunoreactivity was observed in 65 % of the lymphoma tissue samples and in 25 % of the stroma samples and exhibited a nuclear-cytoplasmic type of expression. Dispersed RCAS1-positive macrophages were also identified in the stromata of the lymphoma tissue samples (Fig. 2).

**Fig. 3:**
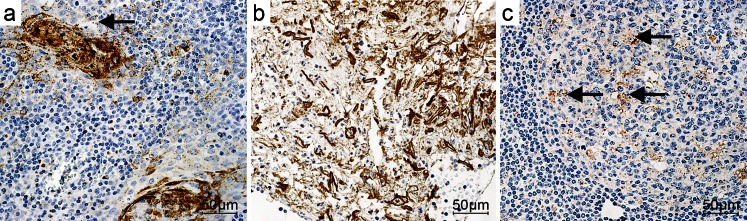
RCAS1 immunoreactivity in chronic tonsillitis. **a** RCAS1 immunoreactivity in the crypt epithelium (*arrow*). **b** RCAS1-positive exfoliated epithelial cells and RCAS1-positive macrophages in the lumen of the crypts. **c** RCAS1 immunoreactivity in the germinal centers (*arrows* RCAS1-positive macrophages). Magnification ×40. *Bars* 50 μm

RCAS1 immunoreactivity was observed in the palatine tonsils of patients with chronic tonsillitis (Fig. 3) and in 60 % of the lymphatic tissue samples as dispersed cells, which were identified as macrophages, in the germinal centers of lymphatic tissue and in all samples of the reticular epithelium of the tonsillar crypts. RCAS1 was not found in the remainder of the tonsillar epithelia. The type of immunoreactivity exhibited was membrane-cytoplasmic. Additionally, RCAS1-positive exfoliated epithelial cells were observed in the crypts of the palatine tonsils and single RCAS1-positive macrophages were seen in the lumen of the crypts.

### Analysis of immunoreactivity of antigens in lymphatic tissue and epithelium lining of tonsils in cases of chronic tonsillitis

The results are presented in Table 3.

**Table 3:** Comparison of antigen immunoreactivity in lymphatic tissue and epithelium of palatine tonsils from patients with chronic tonsillitis (*RCAS1* receptor cancer-binding antigen expressed on SiSo cells)

Antigen	Lymphatic tissue of palatine tonsils under chronic tonsillitis, median (Q_3_-Q_1_)	Tonsillar epithelium, median (Q_3_-Q_1_)	*P*-value
CD3	2	2	0.01
	(0)	(1)	
CD25	2	3	0.05
	(0)	(1,5)	
CD68	3	0	< 0.0001
	(0,5)	(0)	
CD69	3	0	< 0.0001
	(0)	(0)	
Foxp3	3	0	< 0.0001
	(0)	(0)	
RCAS1	1	3	0.0003
	(2)	(2)	

### Analysis of immunoreactivity of antigens studied in lymphoma tissue samples and their microenvironment

The results are presented in Table 4.

**Table 4:** Comparison of immunoreactivity of analyzed antigens in lymphoma tissue samples and their stroma (*NS* not significant)

Antigen	Lymphoma, median (Q_3_-Q_1_)	Stroma, median (Q_3_-Q_1_)	*P*-value
CD3	2	1	0.01
	(0)	(1)	
CD25	1	0	0.01
	(0)	(1)	
CD68	1	0	0.02
	(0)	(1)	
CD69	1	1	NS
	(0)	(1)	
Foxp3	0	0	NS
	(1)	(0)	
RCAS1	1	0	0.02
	(1)	(0)	

The lymphoma tissue samples were characterized by the significantly higher infiltration of CD68-positive macrophages, which were also typified by RCAS1 immunoreactivity, than in the stroma tissue samples. This demonstrates the strong suppressive profile of the tumor tissue in comparison with the microenvironment, as represented by RCAS1-positive macrophages. These cells predominantly infiltrate the tumor tissue but are also present in the microenvironment, thereby determining the suppressive profile of the stroma. This also reflects the tumor-stroma interaction by the expression of these antigens and the presence of RCAS1-positive macrophages. No statistically significant differences were observed in CD69 and Foxp3 antigen immunoreactivity.

### Comparison of analyzed antigen immunoreactivity in lymphoma tissue samples and lymphatic tissue of palatine tonsils

The results are presented in Table 5.

**Table 5:** Comparison of analyzed antigen immunoreactivity in tissue samples of lymphoma and in lymphatic tissue of palatine tonsils

Antigen	Lymphoma, median (O3-Q1)	Lymphatic tissue of palatine tonsils, median (Q_3_-Q_1_)	*P*-value
CD3	2	2	NS
	(0)	(0)	
CD25	1	2	<0.001
	(0)	(0)	
CD68	1	3	<0.001
	(0)	(0.5)	
CD69	1	3	<0.001
	(0)	(0)	
Foxp3	0	3	<0.001
	(1)	(0)	
RCAS1	1	1	NS
	(1)	(2)	

### Comparison of studied antigen immunoreactivity in stroma of lymphoma and epithelium of palatine tonsils (reference group)

The results are presented in Table 6.

**Table 6:** Comparison of immunoreactivity of studied antigens in lymphomatic stroma and in epithelia of palatine tonsils (reference group)

Antigen	Stroma, median (O3-Q1)	Reference group, median (Q_3_-Q_1_)	*P*-value
CD3	1	2	NS
	(1)	(1)	
CD25	0	3	<0.001
	(1)	(1.5)	
CD68	0	0	0.01
	(1)	(0)	
CD69	1	0	<0.001
	(1)	(0)	
Foxp3	0	0	NS
	(0)	(0)	
RCAS1	0	3	<0.001
	(0)	(2)	

## Discussion

In the present study, we demonstrated the presence of RCAS1 protein immunoreactivity in 65 % of the examined tissue samples of DLBCL of the palatine tonsils and in 25 % of the analyzed stromata in which it occurred in CD68-positive cells, which were identified as macrophages and were dispersed throughout the stroma. The immunoreactivity of RCAS1 was statistically significantly higher in the malignant tissue than in the stroma and the number of RCAS1-positive macrophages was higher in the lymphoma than in the stroma. To our knowledge, this is the first investigation concerning the immunoreactivity of RCAS1 in DLBC lymphoma originating in the palatine tonsil and its stroma. The lymphoma cells, like the cells of other types of malignant neoplasms, seem to use the expression of RCAS1 for tumor escape from host immunological surveillance. Moreover, as we have previously discovered, the lymphoma seems to create the suppressive microenvironment by using RCAS1-positive macrophages in order to control the activity of the infiltrating immune system cells. In the literature, Ohshima et al. (2001) demonstrated the expression of RCAS1 in Hodgkin lymphoma; they established that RCAS1 is expressed by malignant Hodgkin and Reed Sternberg cells of EBV-associated Hodgkin disease and that this expression allows these cells to evade the host immune response (Ohshima et al.^2001^). In T-cell leukemia, positive staining for RCAS1 expression has been identified in patients whose survival time is short, whereas in B-cell lymphoma, RCAS1 has only been identified in 1 of 8 cases. RCAS1 has been concluded to be associated with tumor escape from host immunological surveillance, especially in cells infected by the T-lymphotropic virus type I (Muta et al. 2004). The presence of RCAS1 in the tumor is responsible for the process of tumor escape from host immunological surveillance by the inhibition of the growth and the induction of the apoptosis of T and B lymphocytes and NK cells (Sonoda et al. 1996, 2005, 2006, 2008, 2009; Sonoda 2011). RCAS1 can also be shed into blood serum and pleural effusions in a soluble form and can be a useful marker for human cancer and the predictor of the results of the treatment (Sonoda and Kato 2014; Enjoji et al. 2004). In this way, tumor cells use RCAS1 expression for the elimination of cytotoxic immune cells from their own microenvironment and carry out the process of selective immune suppression to create the suppressive microenvironment (Sheu et al. 1997). Physiologically, this mechanism is responsible for the proper regulation of the immune system response and this regulation seems to be disturbed in patients with malignant neoplasms originating from the lymphatic system, such as lymphoma. In our study, RCAS1 immunoreactivity in the lymphoma tissue samples remained at a level comparable with that of the reference group tissue samples; this level was significantly higher in these samples than in those from the stroma. Since the palatine tonsil is a specialized lymphatic tissue organ responsible for creating the immune response, the proper regulation of this process is essential and RCAS1 expression is one of the modes of such regulation. The comparable level of RCAS1 in tissue samples from patients with chronic tonsillitis and in lymphoma samples establishes the existence of a high level of immune cell activity inhibition. In patients with chronic tonsillitis, RCAS1 expression might be induced by bacterial inflammation requiring the restriction of excessive immune cell activity. By contrast, in cases of lymphoma, RCAS1 expression seems to be linked with the loss of local host immune control.

This observation differs from the results obtained from epithelial malignant neoplasms, such as squamous cell carcinoma of the palatine tonsils, in which the expression of RCAS1 has been shown to be significantly higher in the tumor than in the reference group tissue samples (Dutsch-Wicherek et al. 2012). Nevertheless, the detection of this expression in the malignant cells of DLBCL of the palatine tonsils indicates the potential role of RCAS1 protein in the phenomenon of selective immune suppression and the creation of tumor-induced chronic inflammation in this disease, as in other malignant diseases.

The identification of immune cell presence and activity in our study was difficult because of the particular immunohistochemistry method that we used, which allows for the identification of only one antigen. We were therefore unable to identify the precise type of the infiltrating cells. In attempting to interpret this subsequently, we noted that the lymphatic tissue of the palatine tonsils in chronic tonsillitis was characterized by the statistically significantly higher infiltration of CD25-positive cells and higher Foxp3 antigen immunoreactivity than in the lymphoma sample. Because Foxp3 and CD25 are antigens that identify Treg cells (Whiteside 2014), a greater number of these cells are thought to infiltrate the lymphatic tissue of the palatine tonsils in chronic tonsillitis, i.e., under conditions of chronic inflammation. We also found a statistically significantly higher number of CD68-positive cells infiltrating the lymphatic tissue of the palatine tonsils in chronic tonsillitis and a statistically significantly higher number of CD69 antigen-positive cells in the palatine tonsil lymphatic tissue in chronic tonsillitis than in the lymphoma tissue samples. The immune response therefore seems to be stronger in the chronically inflamed tissue than in the lymphoma, which seems to be only sparsely infiltrated by the activated immune cells. The observed differences in the type and intensity of immune infiltrate in the lymphoma and the tissue samples of the reference group (derived from patients with chronic tonsillitis) might reflect the nature of the lymphoma disease with the suppression of the immune system cell activity and the decrease in the number of the immune system cells infiltrating the tumor. A similar tendency was observed in the number and activity of the infiltrating immune system cells between the stroma and the reference group tissue.

In conclusion, chronic inflammation of the palatine tonsils results in intense infiltration by various types of immune system cells and excessive RCAS1 immunoreactivity, both of which confirm the important regulatory role of RCAS1 in the immune response in the mucosa-associated lymphatic tissue of Waldeyer’s ring. RCAS1 seems to be involved in creating the tumor-induced inflammation in the tumor and its microenvironment.
